# Physical exercise and body-mass index in young adults: a national survey of Norwegian university students

**DOI:** 10.1186/s12889-019-7650-z

**Published:** 2019-10-23

**Authors:** Michael Grasdalsmoen, Hege Randi Eriksen, Kari Jussie Lønning, Børge Sivertsen

**Affiliations:** 1 Department of Sport, Food and Natural Sciences, Western Norway University of Applied Sciences, Bergen, Norway; 2 Vestre Viken HF, Drammen, Norway; 3 The Student Welfare Organisation of Oslo and Akershus (SiO), Oslo, Norway; 4 Department of Health Promotion, Norwegian Institute of Public Health, Postboks 973 Sentrum, 5808 Bergen, Norway; 5 Department of Research and Innovation, Helse Fonna HF, Haugesund, Norway; 6 Department of Mental Health, Norwegian University of Science and Technology, Trondheim, Norway

**Keywords:** College students, Physical exercise, Body-mass index, Epidemiology, Trend

## Abstract

**Background:**

Physical inactivity and obesity pose a major public health challenge. The aim of this study was to describe the level of physical exercise and body-mass index in college and university students, as well as to examine potential changes from 2010 to 2018.

**Methods:**

Data stem from the SHoT study, a national student health survey for higher education in Norway, conducted at 4-year intervals. The SHOT studies conducted so far in 2010, 2014 and 2018, included 6053, 13,525 and 50,054 fulltime students (aged 18–35), respectively. Exercise frequency (average number of times exercising each week) was assessed in all three waves, and was used for the trend analysis. The last wave in 2018 also assessed the average intensity and duration of the exercise.

**Results:**

Overall, students exercised less in 2018 compared to 2014, but comparable to level in 2010. The prevalence of overweight increased substantially from 2010 to 2018, but especially in the last 4 years and among older female students. Less than one of four male, and one of five female students, met the recommended criteria for both exercise frequency, intensity and duration. As expected, the associations between exercise and overweight/obesity were in a dose-response manner, and strong across all three waves.

**Conclusions:**

Our findings show that the large majority of young adults fail to meet international recommendations on exercise, and that the proportion of overweight is increasing in both genders and across all age groups. We conclude that there is an urgent need for a broad approach to achieve a paradigm shift in supporting our college and university students to become more active.

## Introduction

Research on overweight and obesity shows that the prevalence has increased substantially across the globe over the last decades. One third of the world’s population can now be categorized as being overweight, and all signs point to a further escalation in the years to come [1]. What may be labelled as the global obesity epidemic is an obvious public health concern, given the many and extensive negative health consequences.

The obesity epidemic can largely be explained by either failure to restrict energy intake, and/or too low levels of energy expenditure. While the relative importance of these factors is still debated [2, 3], there is general agreement that a sedentary lifestyle is one of the most prominent risk factors for the increase in body-mass index (BMI). Despite public health efforts aimed at encouraging individuals to eat healthier and to exercise more, no country has yet been successful in reversing the obesity rates observed during the past 30 years. Public health efforts regarding exercise have included establishing guidelines on the level of physical activity, and provided recommendations and guidance on both the frequency, duration, and intensity of physical activity. For example, the US Centers for Disease Control and Prevention recommend that adults (aged 18–64 years) should get at least 30 min (or preferable 60 min for increased health benefits) of moderate to vigorous physical activity (MVPA) at least 5 days per week [4]. The World Health Organization (WHO) have published very similar recommendation, and have aimed to reduce the prevalence of insufficient physical activity by 10% by 2025 [5]. However, recent evidence show that such efforts have largely been unsuccessful, with a report from the Global Burden of Disease (GBD) study showing that the world’s level of inactivity has not improved from 2001 to 2016 [6]. While our leisure-time activity level has progressively increased over the years [7]; this has not been enough to counterweigh our increased sedentary lifestyle, as our total physical activity level seems to be declining globally [8].

College and university students may be especially at risk for sedentary behaviour, as much of their campus day consists of classroom lectures and studying sitting still. On the other, young adults are typically in good health, and both educational institutions and student welfare associations facilitate their students to engage in various forms of physical activity. Indeed, studies have suggested that university students are both highly sedentary *and* highly active [9, 10]. However, most studies in this field have been conducted on young undergraduate students, often focusing on the transition between late adolescence and young adulthood. Less is known to what extent the association between physical activity and BMI changes with increasing age within the student population. And importantly, there is a paucity of studies investigating if level of physical activity among university students have changed over the decade, and to what extent this may related to the increasing prevalence on overweight and obesity. Expanding our knowledge on these issues in college populations may be especially important, as sedentary behaviour in this age may represents a continued snowballing risk for the development of poor health.

Based on these considerations, the aim of this large national study from 2018 was fourfold: 1) to investigate the current level of physical exercise among male and female college and university students in terms of how many meet the international recommendations of frequency, intensity and duration of exercise; 2) to explore the trend of both physical exercise and BMI from 2010 to 2018; 3) to examine the association between physical activity and overweight across different age cohorts within the student population and 4) to examine the strength of the association between exercise frequency and BMI from 2010 to 2018.

## Methods

### Procedure

The SHoT study (*Students’ Health and Wellbeing Study)* is a large Norwegian student survey for higher education, conducted by three largest student welfare associations. Since 2010, three waves have been completed. Detailed information of the SHoT study has been described in a previous publication [11], but in brief, data from *the SHoT2018* was collected from February to April, 2018, and included all fulltime Norwegian students aged 18–35, taking higher education. In this wave, 162,512 students received an invitation to participate, of whom 50,054 students completed the web-based questionnaires (response rate: 30.8%). *The SHoT2014* study took place from February to March, 2014. In all, 47,514 randomly invited students aged 18 to 35 (stratified by institutions, faculties, and departments) received the online questionnaires, of whom 13,525 students participated (response rate: 28.5%). The first SHoT study, conducted from October to November 2010, were smaller and included 6053 participants of the originally invited 26,779 Norwegian students, also aged between 18 and 35 (response rate of 22.6%).

### Instruments

#### Demographic information

All students provided data on their age and gender. In the current study, the participants’ age was categorized into five groups (18–20 years [18%, *n* = 8832], 21–22 years [31%, *n* = 15,471], 23–25 years [32%, *n* = 15,902], 26–28 years [12%, *n* = 5710], and 29–35 years [7%, *n* = 3427]).

#### Physical activity

The students were first presented with the following brief definition of exercise: “*With exercise we mean that you for example go for a walk, go skiing, swim or take part in a sport*”. Physical activity was then assessed using three sets of questions, assessing the average number of times exercising each week, and the average intensity and average hours each time [12]: 1*) “How frequently do you exercise?”* (Never, Less than once a week, Once a week, 2–3 times per week, Almost every day); 2) *“If you do such exercise as frequently as once or more times a week: How hard do you push yourself?* (I take it easy without breaking into a sweat or losing my breath, I push myself so hard that I lose my breath and break into a sweat, I push myself to near-exhaustion); and 3) *“How long does each session last?”* (Less than 15 min, 15–29 min, 30 min to 1 h, More than 1 h”. This 3-item questionnaire has previously been used in the large population-based Nord-Trøndelag Health Study (the HUNT studies). Previous validation studies [12, 13] have demonstrated moderate correlations between the questionnaire responses and direct measurement of VO_2_max during maximal work on a treadmill (*r* = 0.43[frequency], *r* = 0.40 [intensity] and *r* = 0.31 [duration]), with ActiReg [14, 15], an instrument that measures PA and energy expenditure (EE), and with the International Physical Activity Questionnaire [16].

The item assessing exercise *frequency* was included in all three SHoT studies, whereas the items assessing the *intensity* and *duration* of the exercise were only included in SHoT2018. As a measure of physical activity, the frequency variable was dichotomized using 2 times per week as the cut-off value (inactivity = “never”, “less than once a week”, “once a week”).

Based on WHO’s recommendation [4] that adults (≥18 years) should get at least 30 min (or preferable 60 min for increased health benefits) of MVPA 5 days or more per week (=150 [or preferable 300] minutes per week) [4], two dichotomous recommendation variables were created based on the students’ responses on all three exercise items: 1) MVPA: 150 mins/week: students answering both “Almost every day” on the frequency item, “I push myself so hard that I lose my breath and break into a sweat” on the intensity item, and “30 minutes or more” or “More than 1 hour” on the duration item. 2) MVPA: 300 mins/week: students answering both “Almost every day” on the frequency item, “I push myself so hard that I lose my breath and break into a sweat” on the intensity item, and “More than 1 h” on the duration item.

#### Body mass index (BMI)

BMI was calculated based on self-reported body weight (kg) divided by squared height (m^2^) [17, 18]. The BMI was then split into 4 categories: underweight (BMI < 18.5), normal weight (BMI 18.5–24.9), overweight (BMI 25.0–29.9) and obesity (BMI ≥ 30) [19].

### Statistical analyses

We used IBM SPSS Statistics 25 for Windows (SPSS Inc., Chicago, IL) for all analyses. Independent samples t-tests and Pearson’s chi-squared tests were used to examine differences in physical exercise and BMI in 2010, 2014 and 2018 among male and female students. Multinomial regression models were computed to obtain effect-size estimates of the association between physical exercise and BMI category. Results are presented as odds-ratios (ORs) with 95% confidence intervals (95% CIs), adjusted for gender and age. Missing values were handled using listwise deletion, and there was generally little missing data (*n* < 250 of 50,054 on the three exercise items in SHoT 2018).

## Results

### Physical exercise in SHoT2018

**Fig. 1:**
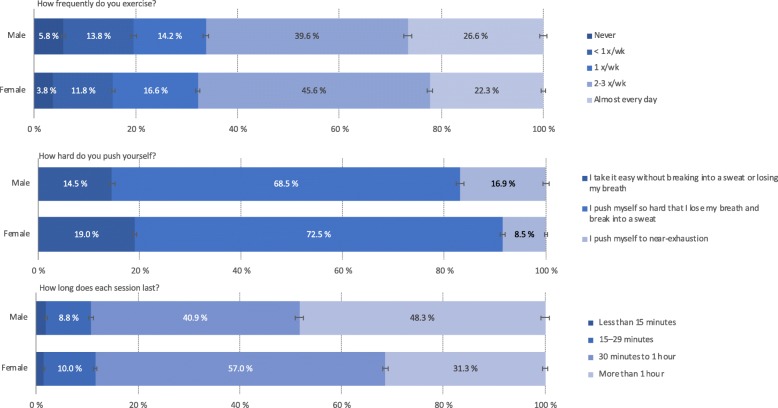
Response pattern of physical exercise in college/university students in the SHoT2018 study. Error bars represent 95% confidence intervals

Approximately two thirds (66% in males and 68% in females) reported exercising twice per week or more frequently, while one in four students (27% in males and 22% in females) reported exercising almost every day. In contrast, 6 and 4% among the male and female students, respectively, reported that they never exercised. In terms of *intensity* of the exercise, the majority of both male (69%) and females (73%) students reported “pushing themselves so hard that they lose their breath and break into a sweat”. More male than female students (17% vs. 9%, *p* < .001) reported “pushing themselves to near exhaustion”. A similar gender difference was also observed for exercise *duration*, with nearly half of the male students (48%) reporting an average exercise duration of more than 1 h, whereas less than one third (31%) of the female students reporting this (see Fig. 1 for details).

In terms of meeting the minimum recommended criteria for both exercise frequency, intensity and duration (150 min per week of MVPA), 23.3% of male and 17.9% of female students, respectively, fulfilled these criteria (gender difference: *p* < .001). Employing the more recent and strict criteria of 300 min per week of MVPA, the corresponding proportions for male and female students were 16.8 and 9%, respectively (*p* < .001).

### Trend of physical exercise from 2010 to 2018

**Fig. 2:**
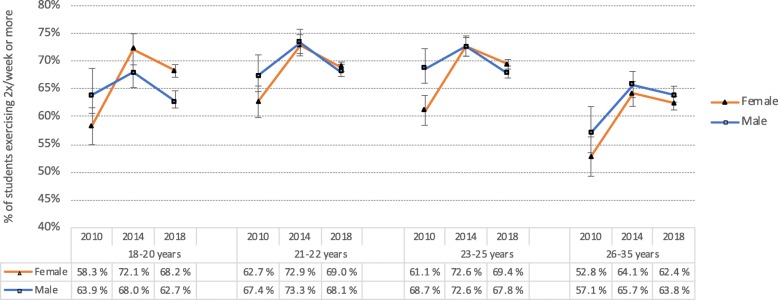
Proportion of college/university students exercising twice or more weekly in 2010, 2014 and 2018 stratified by gender and age group. Error bars represent 95% confidence intervals

There was an overall increase in students exercising twice or more per week from 2010 to 2014 (from 65.1 to 70.4% in males, and from 59.4 to 70.6% in females). However, this trend was no longer present, and to some extent *reversed*, in 2018, with 66.3 and 67.9% of the male and female students, respectively, reporting exercising twice or more per week in 2018. This trend was evident across all age groups (see Fig. 2 for details).

### Trend of BMI and obesity from 2010 to 2018

**Fig. 3:**
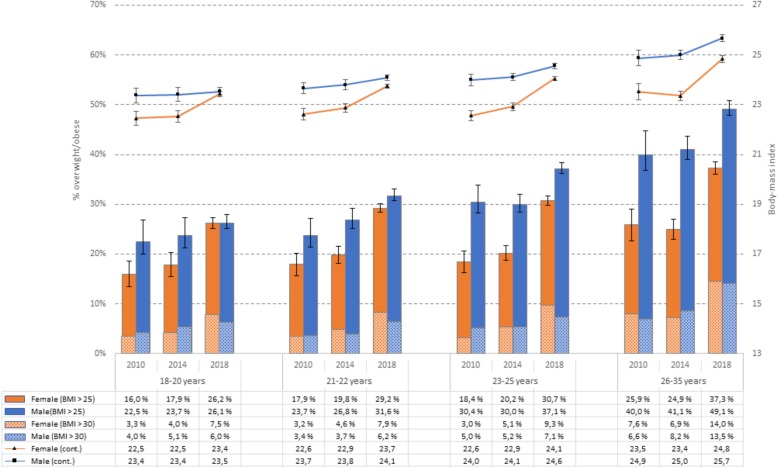
Prevalence of overweight (BMI > 25; solid bars) and obesity (BMI > 30; shaded bars) on left axis, and continuous BMI on right axis among university and college students in 2010, 2014 and 2018 stratified by gender and age group. Error bars represent 95% confidence intervals

There was small increase in BMI among male students from 2010 (24.0 (SD = 3.4) to 2014 (24.2 (SD = 3.5, t(5019) = 2.824, *P* = .002), and the BMI had increased significantly further in 2018 to 24.5 (SD = 3.9, t(10,201.5) = 5.078, *P* < .001). The same pattern was observed in female students: from 22.7 (SD = 2.7) in 2010 to 23.0 (SD = 3.9) in 2014 (t[9021) = 3.338, *P* < .001), and with a substantial increase from 2014 to 2018 (24.0, SD = 4.5, *P* < .001, t(12,020.5) = 19.542, *P* < .001). The increase was evident across all age groups, and as detailed in Fig. 3, the BMI was substantially higher among older students. Of note, there were no significant changes in body height in neither male students (182.2 cm [2010]; 181.9 cm [2014] and 182.2 cm [2018]) nor females students (168.0 cm [2010], 167.8 cm [2014] and 167.9 cm [2018]).

In terms of overweight/obesity, a similar pattern was observed for both men and women. While the prevalence of overweight/obesity in male students was 29% in 2010, the proportion of male students with a BMI over 25 had increased to 31.6% in 2014 and 36.4%in 2018 (*P* < .001). The increase from 2014 to 2018 was even more pronounced in female students. Whereas 19.2% of the female students could be classified as overweight/obese, the prevalence had increased to 20.9% in 2014 and 30.5% in 2018 (*P* < .001). These trends were similar across all age groups (see Fig. 3 for details), although the prevalence of overweight/obesity was substantially higher in the older age groups. For example, in 2018 37.3 and 49.1% of the female and male students, respectively, aged 26–35 years, had a BMI over 25. As also can been seen in Fig. 3 the prevalence of obesity (BMI > 30; displayed in shaded bars), increased in a similar pattern over time, with the strongest increase from 2014 to 2018.

### Association between age, physical exercise and BMI

**Fig. 4:**
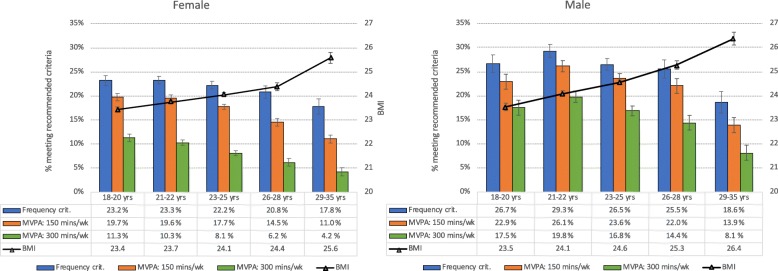
Percentage of male and female students meeting different physical exercise recommendations (bars [left axis]) and BMI (black line on right axis), stratified by age groups

Figure 4 shows the proportion of students meeting the different exercise recommendations against BMI, stratified by gender and age groups. A clear age trend was observed for both male and female students. While about one in four students met the frequency criteria (exercising almost every day) at age 18–20 years, this was reduced to about 18% in students aged 29–35 years. An even stronger age gradient was observed when examining the proportion of students meeting both the *frequency*, *intensity* and *duration* criteria; in females, 20 and 11% of 18–20-years-olds met all three criteria for 150 and 300 min per week of MVPA, respectively, while only 11 and 4%, respectively, of the oldest age group did this. A similar decline was observed in males regarding the two exercise recommendations, from 23 and 18% in 18–20-year-olds, to 14 and 8% in 29–35-year-olds. This general decline in level of physical activity in older students was inversely associated with BMI, for which a significant increase with advancing age was observed (see Fig. 4 for details).

### Association between exercise frequency and BMI from 2010 to 2018

Across all three time points, exercise frequency was associated with increased odds of being obese in a graded/dose-response manner; the less frequent his or she exercised, the higher the odds of having a BMI over 30. For example, in SHoT2018, exercising once per week was associated with an OR of 2.22 of being obese, whereas the OR increased to 3.29 in student never exercising, compared to exercising almost every day. Similar patterns were observed also in 2010 and 2014. In terms of odds of being overweight, a similar graded association was observed in 2010, but not in 2014 and 2018 (see Table 1 for details).

**Table 1:** Odds-ratios of frequency of physical exercise (exposure reference category: *Almost every day*) associated with BMI category (outcome reference category: *normal weight*) among Norwegian university students in 2010, 2014 and 2018. ORs adjusted for age and gender

Exercise frequency	Normal weight (reference)		Overweight (BMI 25.0–29.9)				Obesity (BMI ≥ 30)			
	%	(n)	%	(n)	OR	95% CI	%	(n)	OR	95% CI
2018										
Never	3.8%	(1191)	3.9%	(442)	1.12	0.99–1.26	7.0%	(299)	3.29***	2.82–3.84
< 1 x/wk	11.0%	(3419)	12.7%	(1440)	1.28***	1.18–1.38	18.8%	(805)	3.00***	2.68–3.36
1 x/wk	15.1%	(4684)	16.3%	(1850)	1.22***	1.14–1.31	19.0%	(814)	2.22***	1.98–2.48
2–3 x/wk	44.4%	(13797)	44.3%	(5044)	1.14**	1.08–1.21	40.9%	(1749)	1.63***	1.48–1.79
Almost every day (ref)	25.7%	(7990)	22.8%	(2599)	1.00	–	14.2%	(606)	1.00	–
2014										
Never	11.4%	(1011)	12.9%	(323)	1.13	0.92–1.40	20.2%	(136)	2.79***	1.83–4.23
< 1 x/wk	15.8%	(1395)	17.9%	(447)	1.18	0.96–1.44	22.8%	(154)	2.32***	1.54–3.51
1 x/wk	42.1%	(3726)	39.0%	(975)	0.99	0.82–1.18	39.0%	(263)	1.52*	1.02–2.26
2–3 x/wk	23.6%	(2090)	23.0%	(576)	1.02	0.84–1.23	13.8%	(93)	0.98	0.63–1.50
Almost every day (ref)	7.1%	(631)	7.2%	(180)	1.00	–	4.2%	(28)	1.00	–
2010										
Never	14.0%	(593)	16.8%	(184)	1.98***	1.33–2.94	25.8%	(60)	3.94***	1.60–9.71
< 1 x/wk	20.9%	(887)	23.1%	(253)	1.86***	1.27–2.74	29.2%	(68)	3.03***	1.23–7.42
1 x/wk	42.1%	(1785)	41.6%	(456)	1.65**	1.14–2.40	34.8%	(81)	1.81	0.74–4.40
2–3 x/wk	17.9%	(757)	15.2%	(166)	1.34	0.90–1.99	8.2%	(19)	1.00	0.38–2.65
Almost every day (ref)	5.0%	(213)	3.3%	(36)	1.00	–	2.1%	(5)	1.00	–

*** *p* < .001; ** *p* < .01; * *p* < .05;

## Discussion

This large national survey from 2018, inviting all fulltime Norwegian university and college students aged 18–35, has several notable findings. While two of three students in 2018 exercised twice or more per week, less than one of four male, and less than one of five female students, met the minimum recommended criteria for both exercise frequency, intensity and duration (150 min per week of MVPA). Overall, students exercised less in 2018 compared to 2014, but comparable to the levels in 2010. The prevalence of overweight increased substantially from 2010 to 2018, but especially in the last 4 years and among older female students. As expected, the associations between exercise and BMI were in a dose-response manner, and strong across all three waves.

### Comparisons to previous studies

Inactivity is a well-recognized risk factor for developing or exacerbating a range of non-communicable diseases (NCS), and a range of studies worldwide have examined the prevalence of inactivity (i.e doing less than 150 min of MVPA per week). In a recent Lancet paper from 2018 including 1.9 million participants from 358 surveys and 168 countries, the authors reported a global age-standardised prevalence of insufficient physical activity of 27.5% (23.4% in men and 31.7% in women), but with great regional differences [6]. Compared to these pooled analyses, the findings from the current 2018 study of students taking higher education in Norway, indicate that a substantially larger proportion fail to meet the recommended criteria of PA. However, we cannot disregard the possibility that some of these differences may be due to methodological issues, including slightly different operationalizations of both frequency, intensity and duration of physical exercise. As such, future studies are needed to verify both if and why the large majority of college and university students fail to meet the recommended criteria for physical activity.

In contrast to the many prevalence reports published on physical activity/inactivity, consistent data on trends of adult physical activity over time have been scarce, and to the best of our knowledge, no previous epidemiological trend studies have been conducted on college and university populations. Although the current study found a small increase in level of physical activity from 2010 to 2014, the overall proportion of inactivity remained relatively stable from 2010 to 2018, which is also in line with the conclusion from the Lancet report focusing on the general adults population [6].

Whereas the prevalence of inactivity have remained stable over the last decade, virtually all available data worldwide show that both the average BMI and proportion of overweight/obesity have increased over the last four decades, among children, adolescents, and especially adults [20]. Our findings are in line with these data, showing a stable increase in BMI and proportion of overweight/obesity in young adults from 2010 to 2018. And while the increase was evident in both male and female students, we found that the prevalence of overweight and obesity had especially increased in older students, aged 26–35. These age and gender-specific findings are also similar to previous Norwegian trend data based in the large population-based HUNT study [21]. And while the HUNT study comprises participants aged 20–89 years of age, the authors of that study found that the largest increase of obesity from 1984 to 1986 to 2006–2008 was observed in the youngest age groups (20–40 years). The current study extends on these findings, and provides further evidence of no halt in the increase of obesity. If this trend continues at a similar pace, it will not be long until the majority of particularly older students (> 25 years) can be classified as overweight or obese.

### A global problem

Despite the steadily escalation of observed weight gain over the last 4 decades, no country have successfully managed to stop or reverse this trend. As a simple explanation of weight gain, the concept of thermodynamics is are often used, with *energy in* minus *energy out* equal to weight profit or loss. Both these mechanisms seem to working negatively as countermeasures stop the weight gain. On one hand, the increased living standards and higher welfare is associated with more sedentary behaviour, a trend which also is also observed in low-income countries, characterized by urbanization, change in food culture and less more quiet-sitting.

### Public health implications

The findings have notable public health implications, as they call attention to both the increasing prevalence of overweight and obesity over the last decade among students taking higher education. As the majority of these young adults failed to meet the recommended levels of frequency, duration and intensity of weekly exercise, our findings have obvious implications both from a public health perspective, but especially for the student welfare associations and educational institutions. Although these institutions and associations often encourage and facilitate their students to take part in a wide range of sports and exercise, our results indicate that they need to increase their efforts. For example, in terms of campus planning, more awareness is warranted regarding the need for good cycle paths and walkways. Another potential approach may be to adopt strategies from e.g. high schools, where physical exercise is a more integrated element in the typical school day, Although there is limited evidence of the effectiveness of broad awareness campaigns to increase the public’s physical activity, there are studies suggesting that various forms of personalized media messages can be used to raise awareness, increase knowledge, and motivate a population to be more physically active.

In response to the alarming levels of inactivity, the World Health Organization (WHO) published in 2010 a set of recommendations to national policy makers on the total amount of physical activity needed to prevent noncommunicable diseases (NCDs), including overweight and obesity. Our findings suggest both that the large majority of young adults still fail to meet these recommendations, and that the recommendations are not sufficient to reduce the obesity epidemic. This dilemma shows that a broader approach is warranted, and the WHO recently launched a global action plan [22] on physical activity for 2018 to 2030 to make people more active. This action plan aims at providing a system-based framework of effective policy actions to countries in order to increase physical activity at all levels, emphasizing the need for a much broader response to achieve a paradigm shift in supporting all people being regularly active. This new WHO campaign will hopefully also be adopted regionally and locally by all educational institutions, and the next wave of the SHoT study (scheduled for 2022), will provide new insight in if the negative trend is possible to stop,

Another important finding in the current study is that female students were particularly at risk of being inactive and be classified as overweight. As such, increased efforts should be made to promote and create more tailored participation possibilities for female students. One option would be to focus more on raising the profile of women’s sport in general, and to make the sporting environments to become more inclusive of women.

### Methodological considerations

First, an important study limitation is the relatively low response rates for all three SHoT waves. But in contrast to similar cohort studies, especially of young adults, who have found *lower* response rates in recent years [23], the SHoT studies’ response rates have actually *increased* from 2010 to 2018. Nevertheless, the response rate of the SHoT2018 was only 31%, and as such, care should be taken when generalizing to the whole student population. One possible reason for the modest response rates may be the use of solely electronic survey platforms, which generally yield lower participation rates than for example paper-based surveys or face-to-face interviews [24]. Second, another limitation is that the three data collection methods were not completely identical. Although all three waves were web-based and included several of the same questionnaires, both the graphical user interface and months of data collection differed somewhat, which should be taken into account especially when examining trend results. A third study limitation is related to the physical activity measure, as it is more accurate to say that we assessed *perceived* intensity, as less fit individuals will feel exhausted by an intensity that a fit person will feel comfortable. Similarly, there may be other combinations of MVPA that can be performed to meet the recommended levels of physical activity. For example, 150 min of MVPA can be replaced with a lower duration of higher intensity, which are not accounted for in the current study. A fourth limitation is that while we did include a detailed assessment of energy *expenditure*, the SHoT study did not assess energy *intake*, which of course is vital when exploring the trend of obesity. Finally, there are limitations related to the use of BMI, as it measures excess weight rather than excess fat. BMI does not distinguish between excess fat, muscle, or bone mass, nor does it provide any indication of the distribution of fat among individuals. The strengths of the SHoT study include the very large sample size, in combination with several well-validated questionnaires.

## Conclusion

The current study shows that that the large majority of young adults fail to meet international recommendations on exercise, and that the proportion of overweight is increasing in both genders and across all age groups. There is an urgent need for a broad approach to achieve a paradigm shift in supporting our college and university students to become more active, a responsibility that lies with both political and education institutions, as well as the student welfare associations.

## Data Availability

The SHoT dataset is administrated by the NIPH. Approval from a Norwegian regional committee for medical and health research ethics [https://helseforskning.etikkom.no] is a pre-requirement. Guidelines for access to SHoT data are found at [https://www.fhi.no/en/more/access-to-data].

## Abbreviations

BMI: Body-mass index
MVPA: Moderate to vigorous physical activity
OR: Odds-ratio
PA: Physical activity
SHOT: Students’ Halth and Wellbeing Study
WHO: World Health Organization
